# Prevalence and association of Epstein-Barr virus infection with sinonasal inverted papilloma and sinonasal squamous cell carcinoma in the northeastern Thai population

**DOI:** 10.1186/s13027-020-00308-5

**Published:** 2020-06-24

**Authors:** Thawaree Nukpook, Tipaya Ekalaksananan, Watchareporn Teeramatwanich, Natcha Patarapadungkit, Surachat Chaiwiriyakul, Patravoot Vatanasapt, Sirinart Aromseree, Chamsai Pientong

**Affiliations:** 1 Department of Microbiology, Faculty of Medicine, Khon Kaen University, Khon Kaen, Thailand; 2 HPV & EBV and Carcinogenesis Research Group, Khon Kaen University, Khon Kaen, Thailand; 3 Department of Otorhinolaryngology, Faculty of Medicine, Khon Kaen University, Khon Kaen, Thailand; 4 Department of Pathology, Faculty of Medicine, Khon Kaen University, Khon Kaen, Thailand

**Keywords:** Epstein-Barr virus, Sinonasal inverted papilloma, Sinonasal squamous cell carcinoma, Inflammation

## Abstract

**Aims:**

Sinonasal inverted papillomas (SIP) and sinonasal squamous cell carcinomas (SNSCC) are sinonasal tumors with unclear etiology and pathogenesis. Epstein-Barr virus (EBV) has been detected in these tumors but information concerning their association is still limited. This study aimed to investigate the prevalence in, and association of EBV infection with SIP and SNSCC in northeastern Thailand.

**Methods:**

DNA was extracted from 226 formalin-fixed, paraffin-embedded tissues including 80 nasal polyps (NP; the control group), 64 SIP and 82 SNSCC samples. Presence of EBV in these tissues was investigated using real-time PCR and their localization within tissues was confirmed using in situ hybridization (ISH). Characteristics of patients and the association of EBV prevalence with sinonasal tumors were analyzed.

**Results:**

SIP and SNSCC were frequently found in people aged > 50 years and more often in males than in females (3:1 ratio). EBV infection was detected in 33.75, 64.06 and 37.80% of NP, SIP and SNSCC tissues, respectively, by real-time PCR. There was a statistically significant association between EBV infection and SIP (odds ratio [OR] = 3.52). This was not the case for SNSCC when compared to the NP group (OR = 1.83). Interestingly, EBV infection tended to be associated with inflammation and dysplasia in SIP. In SNSCC, EBV was mostly found in samples with undifferentiated or poorly differentiated cell types as well as in recurrent cases and lymph-node metastasis. Using ISH, EBV was detected only in infiltrating lymphocytes within the tumor stroma, not in the tumor epithelial cells.

**Conclusions:**

Infiltrating lymphocytes containing EBV in the tumor microenvironment might enhance tumorigenesis of SIP and SNSCC. The mechanism by which EBV promotes development of SIP and SNSCC needs to be elucidated in the future.

## Background

Epstein-Barr virus (EBV) is a human herpesvirus 4 present in more than 90% of the world’s population. The primary infection with EBV in B lymphocytes may cause a short-term proliferation of infected B cells, and then latent infection in memory B cells, but infection with EBV is self-limited by the host immune response, mostly involving T cells [1]. EBV is the causative agent of infectious mononucleosis, mainly seen in adolescents and young adults. In addition, various types of cancers have been associated with EBV infection such as Burkitt’s lymphoma, Hodgkin lymphoma, oral cancer, nasopharyngeal carcinoma, breast cancer, gastric cancer and hepatocellular carcinoma [2–5]. EBV may also trigger chronic inflammation and contribute to tumor formation by modulation of cell proliferation and inhibition of apoptosis [6].

Sinonasal inverted papilloma (SIP) is a benign tumor of the nasal cavity and paranasal sinuses [7, 8]. It is seen three times more frequently in males than in females at the fifth and sixth decade of life [9]. The term “inverted papilloma” describes the epithelial growth inward into the underlying supportive tissue of the nasal cavity and paranasal sinuses that is characteristic of the tumor [10]. It is the second most common benign tumor of the sinonasal tract, representing approximately 0.5–4% of all primary nasal tumors. SIP is well known for its invasiveness, high recurrence rate and association with sinonasal squamous cell carcinoma (SNSCC) [11]. The etiology of SIP remains unclear. Several hypotheses have been proposed but none has gained general acceptance [12]. Due to rarity of this disease, there is limited information on its characteristics and treatment outcomes. Human papillomavirus (HPV) and EBV genomes have been reported in SIP tissues and may have some role in SIP etiology. An association between EBV infection and SIP has been reported in a few studies [13].

Sinonasal carcinoma is a cancer of the nasal cavity and paranasal sinuses. It is rare, representing approximately 3–5% of head and neck cancers [14, 15]. Histopathologically, sinonasal carcinomas are subdivided into squamous cell, lymphoepithelial, adenocarcinoma, sinonasal undifferentiated, salivary gland-type and neuroendocrine categories. Squamous cell carcinoma (SCC) is the most common type of cancer of the nasal cavity [16, 17]. Sinonasal squamous cell carcinoma (SNSCC) is best known from East Asia but is nevertheless uncommon and little is known about its etiology and malignancy. Chemical exposure, smoking and oncogenic virus infection are considered to be important risk factors [17]. EBV infection in some histological subtypes of sinonasal carcinoma suggests a role for this virus in the pathogenesis of these carcinomas and presence of EBV in SNSCC is associated with lymph node or distant metastases [18].

EBV is associated with carcinogenesis due to its ability to promote cell growth and inhibit apoptosis, as well as to facilitate cell survival and immune evasion. Accordingly, we hypothesized that EBV infection might be significantly involved in development and progression of SIP and SNSCC. Currently, the prevalence of EBV and its association with SIP and SNSCC are not clearly known in Northeastern Thailand. We therefore gathered information about EBV in three types of nasal tumor; nasal polyps (NP), SIP and SNSCC by using real-time PCR and in-situ hybridization. The association of EBV infection with SIP and SNSCC were assessed by comparison of EBV prevalence with the clinical characteristics of the patients.

## Methods

### Tissue samples and patient characteristics

Tissue samples of nasal polyps (NP), sinonasal inverted papillomas (SIP) and sinonasal squamous cell carcinomas (SNSCC) from 2010 to 2016 were retrieved from archival paraffin blocks stored in the Department of Pathology, Faculty of Medicine, Khon Kaen University. The histological diagnosis was reviewed in each case and confirmed by 2 pathologists. In total, 226 retrospective formalin-fixed paraffin-embedded (FFPE) samples were collected, including 64 SIP, 82 SNSCC and 80 NP, the last used as a control group. Patient characteristics were obtained from surgical and clinical reports. The study was approved by the Khon Kaen University ethics committee in human research.

### DNA extraction

The FFPE sinonasal tissue samples were cut into 4–5 μm sections. Five sections from each sample were used for DNA extraction. Tissue sections were deparaffinized by adding 1000 μl of xylene into the sections and vortexing for 30 s, then removing the xylene by centrifugation at 13,000 rpm for 5 min (3 times). Deparaffinized tissues were washed twice with 95% ethanol: 1000 μl of ethanol were added and vortexed for 30 s, then centrifuged at 13,000 rpm for 5 min. The tissue pellets were then washed with distilled water and air dried. DNA was extracted using a commercially available system, DNeasy blood and tissue kits (Qiagen, Hilden, Germany), according to the manufacturer’s instructions. The extracted DNA was quantified by GAPDH amplification using SYBR green real-time polymerase chain reaction (real-time PCR). All GAPDH-detectable samples were used as subjects for EBV detection by real-time PCR.

### EBV detection by real-time PCR

EBV DNA detection was performed by SYBR green-based real-time PCR using sets of primers specific for two genes: BALF5 and BZLF1 [BALF5: (F) 5′- GGA GAA GGT CTT CTC GGC CTC -3′, (R) 5′- TTC AGA GAG CGA GAC CCT GC-3′ [19, 20]; and BZLF1: (F) 5′-TGT TTC AAC CGC TCC GAC TG -3′, (R) 5′- GGG TTA TGT CGG AGA CTG GG -3′] [21]. The GAPDH gene served as an endogenous control to guarantee the DNA quality and was detected using specific primers (GAPDH (F): 5′ TCA TCA GCA ATG CCT CCT GCA-3′ and GAPDH (R): TGG GTG GCA GTG ATG GCA-3′) [20]. All reactions were performed in a final volume of 20 μl containing 1X SsoAdvancedTM Universal SYBR® Green Supermix (Bio-Rad, Hercules, CA, USA), 0.2 μM of each forward and reverse primer and 50 ng of DNA template. The real-time PCR for EBV DNA was performed using an Applied Biosystems 7500 flats system: cycling conditions started with 3 min at 95 °C followed by 40 cycles of 95 °C for 10 s, 64 °C for 10 s and 72 °C for 30 s. Cycling conditions for the GAPDH gene started with 3 min at 95 °C followed by 40 cycles of 95 °C for 10 s, 58 °C for 10 s and 72 °C for 30 s. DNA from B95–8 (EBV-positive cell line) and SiHa cells were used as the positive control for EBV DNA and the GAPDH gene, respectively.

### Epstein–Barr virus-encoded small RNA in-situ hybridization (EBER-ISH)

To confirm the location of EBV in tumor tissues, after real-time PCR was done, Epstein–Barr virus-encoded small RNA (EBER) was detected using in situ hybridization. FFPE tissue samples were cut into 3 μm sections and mounted on slides. A section of FFPE nasopharyngeal carcinoma tissue was used as the positive control. EBER PNA probe/FITC (DakoCytomation, Y5200, Glostrup, Denmark) and the Ab93705-mouse and rabbit specific HRP/AEC (ABC) Detection IHC Kit (Abcam, Boston, MA, USA) were used as a probe and detection system, respectively. After the tissue samples were deparaffinized, rehydrated, hydrogen peroxide block solution was applied. The EBER probe was applied to the tissue sections and incubated at 55 °C for 90 min followed by rabbit anti-FITC and biotinylated goat anti-polyvalent. Then streptavidin peroxidase was applied followed by AEC Single solution and counterstained with hematoxylin. A red color (positive) signal of EBV-infected cells could be observed under a light microscope and the EBV localization in the tumor tissue was confirmed by a pathologist.

### Statistical analysis

Statistical analyses of EBV prevalence was assessed chi-squared tests and Fisher’s exact tests in GraphPad Prism 5. The correlation between EBV infection and clinical characteristics was done by multiple logistic regression using STATA. Differences were considered to be statistically significant when the *P*-value was ≤0.05.

## Results

### Patient characteristics

A total of 226 tissue samples from NP, SIP and SNSCC patients diagnosed and treated surgically at otolaryngology outpatient clinics, Srinagarind Hospital, Faculty of Medicine, Khon Kaen University, during the period 2010 to 2016 were retrieved from archival paraffin blocks stored in the Department of Pathology, Faculty of Medicine, Khon Kaen University. Characteristics of the patients are listed in Table 1. Nasal tumors were found in individuals of all ages, but most frequently in the fifth to the seventh decades of life and mostly in male (3:1 male-to-female ratio). Statistical analysis using multiple logistic regression shown that age was the only factor significantly associated with SIP and SNSCC. Risks of SIP and SNSCC development increased 3.86 times (OR = 3.86; CI = 1.82–8.19) and 6.43 times (OR = 6.43; CI = 3.11–13.33), respectively, in people aged ≥50 years compared to younger people (Table 2).

**Table 1:** Characteristics of patients and histological findings

Characteristics	NP	SIP	SNSCC
**Number of cases**	80	64	82
**Age (years)**			
< 50	47 (58.8%)	17 (26.6%)	16 (19.5%)
≥ 50	32 (40.0%)	47 (73.4%)	66 (80.5%)
No clinical data	1 (1.2%)	0 (0.0%)	0 (0.0%)
**Sex (%)**			
Male	50 (62.5%)	45 (70.3%)	55 (67.1%)
Female	29 (36.3%)	19 (29.7%)	27 (32.9%)
No clinical data	1 (1.2%)	0 (0.0%)	0 (0.0%)
**Inflammation (%)**			
Sub-acute	0 (0.0%)	2 (3.1%)	
Chronic	15 (18.8%)	2 (3.1%)	
No inflammation	64 (80.0%)	52 (81.3%)	
No clinical data	1 (1.2%)	8 (12.5%)	
**Dysplastic change (%)**			
Focal dysplasia		2 (3.1%)	
Mild dysplasia		2 (3.1%)	
Severe dysplasia		2 (3.1%)	
No dysplasia		50 (78.2%)	
No clinical data		8 (12.5%)	
**Cell differentiation (%)**			
Undifferentiated			1 (1.2%)
Poorly differentiated			4 (4.9%)
Moderately differentiated			12 (14.6%)
Well differentiated			14 (17.1%)
No clinical data			51 (62.2%)
**Invasion (%)**			
Invasive			13 (15.8%)
Not invasive			65 (79.2%)
No clinical data			4 (4.9%)
**Recurrence (%)**			
Yes		3 (4.7%)	8 (9.7%)
No		53 (82.8%)	70 (85.4%)
No clinical data		8 (12.5%)	4 (4.9%)
**Metastasis (%)**			
Yes			1 (1.2%)
No			77 (93.9%)
No clinical data			4 (4.9%)

**Table 2:** The relationship between characteristics of patients and risk of sinonasal tumor

Characteristics	Compared to NP					
	SIP			SNSCC		
	n	OR (95%CI)	***P*** value	n	OR (95%CI)	***P*** value
**Number of cases**	64			82		
**Age (years)**						
< 50	17 (26.6%)	3.86 (1.82–8.19)	0.000	16 (19.5%)	6.43 (3.11–13.33)	0.000
≥ 50	47 (73.4%)			66 (80.5%)		
**Sex (%)**						
Male	45 (70.3%)	0.57 (0.26–1.25)	0.158	55 (67.1%)	1.03 (0.49–2.15)	0.933
Female	19 (29.7%)			27 (32.9%)		
**EBV infection**						
Yes	41 (64.1%)	3.52 (1.70–7.28)	0.001	31 (37.8%)	1.83 (0.89–3.78)	0.100
No	23 (35.9%)			51 (62.2%)		

### EBV DNA in sinonasal tumors

**Fig. 1:**
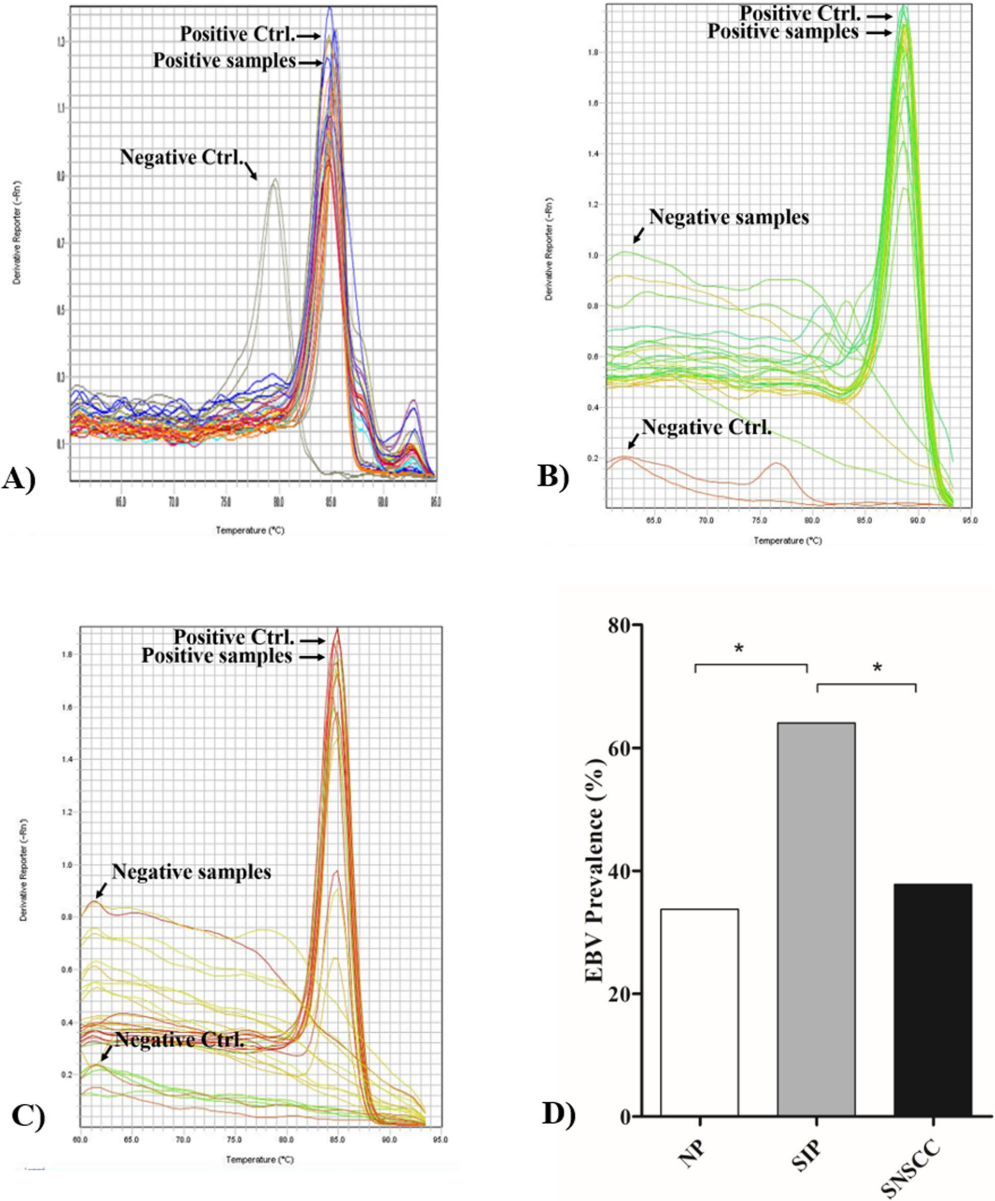
Prevalence of EBV infection in nasal polyps (NP), sinonasal inverted papillomas (SIP), and sinonasal squamous cell carcinomas (SNSCC) according to results of SYBR green real-time PCR. **a**-**c** melting curves of GAPDH, BALF5, and BZLF1, respectively, of positive-, negative-control, and tested samples. **d** EBV prevalence in the tested population

**Fig. 2:**
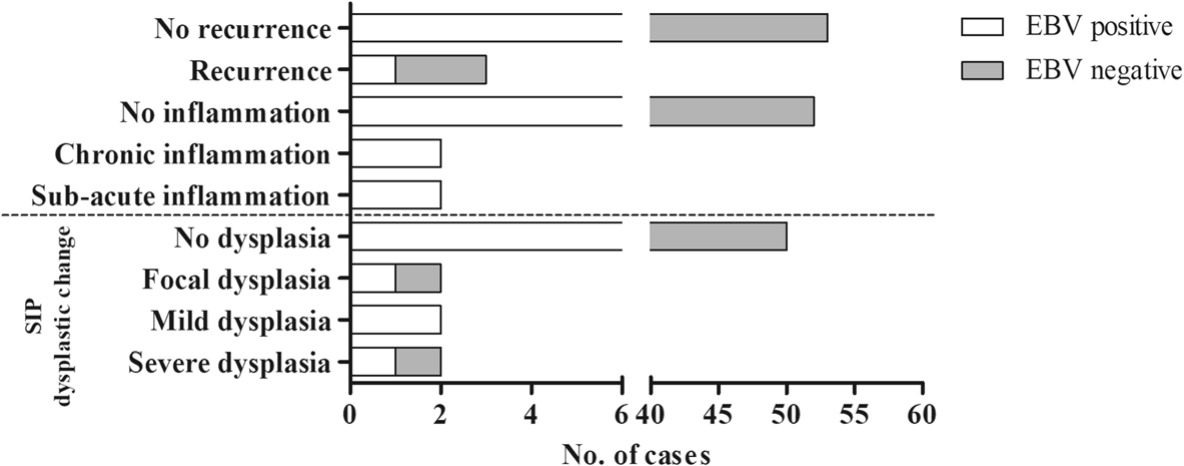
Correlation between EBV infection and clinical characteristics of SIP tissues

**Fig. 3:**
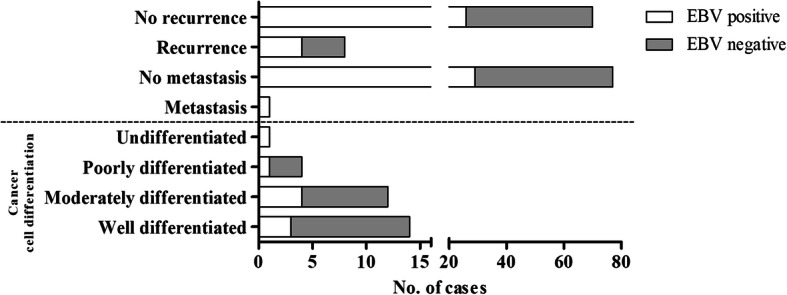
Correlation between EBV prevalence and clinical characteristics of SNSCC tissues

In this study, EBV DNA and the GAPDH gene were detected by SYBR green real-time PCR using specific primers. GAPDH was detected in all samples with a Tm in GAPDH-positive samples of 84.8–85.4 °C: the Tm of the negative control was 79.5 °C (Fig. 1a). Tm values for BALF5 and BZLF1 in EBV-positive samples were 88.5–89 and 84.6–85 °C, respectively. The corresponding values for negative samples were 62 and 61.7 °C, respectively (Fig. 1b-c). EBV DNA was detected in 27/80 (33.75%), 41/64 (64.06%) and 31/82 (37.80%) of NP, SIP and SNSCC cases, respectively (Fig. 1d). EBV infection was significantly more prevalent (*p* < 0.001) relative to the NP control group (Fig. 1d) and positively associated with SIP (OR = 3.52; CI = 1.70–7.28, RR = 1.99; CI = 1.35–2.95). In SNSCC, there was a non-significant increase in prevalence relative to the NP group (OR = 1.83; CI = 0.89–3.78, *RR* = 1.20; CI = 0.88–1.62). The relationship between EBV infection and severity of histopathology classification in SIP was analyzed. This showed that EBV was commonly found in SIP cases with sub-acute inflammation (2/2) and chronic inflammation (2/2), and also found in SIP with dysplasia. EBV was detected in 1/2 (50%), 2/2 (100%), and 1/2 (50%) of SIP samples with focal, mild and severe dysplasia, respectively (Fig. 2). These results suggested that EBV infection is associated with SIP and might be a risk factor for development and progression of SIP. In SNSCC cases, EBV was mostly found in samples with undifferentiated cell types (1/1, 100%), poorly differentiated (1/4, 25%), and moderately differentiated cell types (4/12, 33%): prevalence was relatively low in SNSCC samples with well-differentiated cell types (3/14, 21.4%) as shown in Fig. 3. In addition, EBV was found in 4/8 (50%) of SNSCC recurrent cases and in the single case of lymph-node metastasis, as shown in Fig. 3.

### EBV localization in sinonasal tumors

**Fig. 4:**
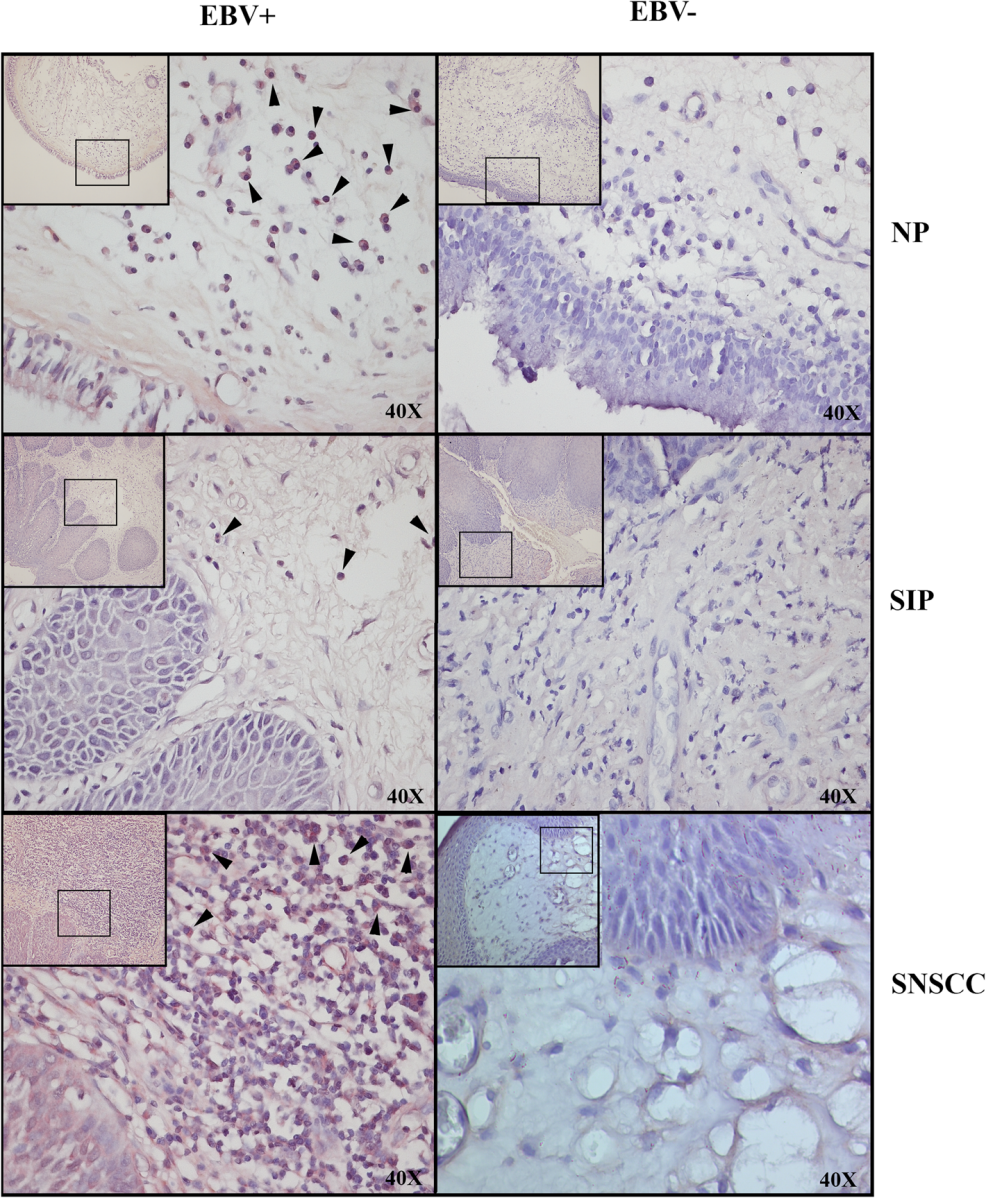
EBV in sinonasal tumor tissues was detected by EBER-ISH. A positive signal (red color) was observed in infiltrating leucocytes within the tumor stroma (black arrows) but not in the tumor epithelium of the EBV DNA-positive sample group in all tumor types. No positive signal was found in the EBV DNA-negative group

To detect EBV in tumor tissues and investigate whether EBV infection occurs in the tumor epithelium or in infiltrating lymphocytes, EBER-ISH was performed. EBER-positive signals were found in all EBV DNA-positive cases, but not in EBER-negative control and EBV DNA-negative cases (Fig. 4). The EBER-positive signals were only found in infiltrating lymphocytes within the tumor stroma, not within tumor epithelial cells.

## Discussion

SIP and SNSCC are nasal tumors of unclear etiology and poorly understood pathogenesis. Several studies have attempted to correlate viral infection with SIP pathogenesis, because various human DNA viruses are known to be associated with tumor development [7]. Previous studies have reported that HPV-6 and HPV-11 are associated with SIP, while HPV-16 and HPV-18 are associated with SIP exhibiting malignant transformation and with SNSCC [22]. However, the role of HPV infection in pathogenesis of SIP remains controversial [12, 23]. The role and association of EBV in SIP pathogenesis is also unclear. EBV prevalence in SIP ranges from 0 to 65% [12, 24]. High prevalence and significant association of EBV infection with SIP was found only in the study by Macdonald and colleagues in 1995, who used PCR to detect EBV DNA in 65% of SIP cases but in no nasal polyp control samples. They concluded that there was a clear association of EBV with SIP [25]. This is consistent with our result: we found a high prevalence of EBV in SIP cases (41/64; 64.06%) and SNSCC cases (31/82; 37.80%) using SYBR green real-time PCR. Others have found low or no EBV infection in NP and SIP tissues using the ISH method [12]. However, we found a rather high prevalence of EBV (27/80; 33.75%) in NP cases, a figure comparable with that of Zaravinos and colleagues [26] who used PCR to detect EBV DNA in 35% of NP samples. This was a significantly higher proportion than in samples from adjacent turbinates used as the control group and supported the hypothesis that EBV infection influences the pathogenesis of nasal polyps [26]. We found that EBV prevalence was significantly higher in SIP compared to SNSCC samples but was non-significantly higher in SNSCC compared to NP samples. Moreover, EBV was commonly found in SIP cases with chronic inflammation and with dysplasia, as well as in SNSCC samples with abnormal differentiated cell types. EBER-ISH demonstrated that only a few infiltrating cells located in tumor stroma in all categories were EBV-positive. These results suggest the involvement of EBV infection in inflammatory processes in NP, SIP, and SNSCC, possibly affecting the pathogenesis of these tumors.

Chronic inflammation is possible etiological factor for SIP, but its pathogenic role here is not well understood. SIP develops from the lateral nasal wall where chronic inflammatory changes are common [7]. A new concept of SIP pathogenesis has been suggested by Lanza and others [27, 28]. They proposed that SIP may be an end stage of a chronic inflammatory condition rather than a true neoplasm. In support of this, they observed an association between sinus inflammation and inverted papilloma [27]. A significantly higher proportion of inflammatory cells was found in SIP compared to other sinonasal papillomas, especially in grade I and II tissues rather than in grades III and IV [28]. Predominant among these inflammatory cells were neutrophils, macrophages, eosinophils, CD8+ T cells and T-reg cells [29]. All of these findings point to chronic inflammation as an important factor in creating a microenvironment to influence abnormal growth of nasal cells [29]. It is well known that EBV is associated with inflammation. EBV components such as latent membrane protein 1 (LMP1) and EBERs activate inflammatory cytokines within infected cells and are associated with malignant progression in several tumors. EBV activates the inflammatory responses by triggering the infected cell to release inflammatory mediators (chemokines and cytokines) into the tumor environment. These mediators recruit inflammatory cells to the site of infection where they are activated to release proinflammatory factors.

In nasopharyngeal carcinoma (NPC), LMP1 induces CXCL10 expression, upregulates IL-8, macrophage inflammatory protein (MIP)-1 α and MIP-1 β to promote lymphocyte infiltration [30, 31]. Moreover, LMP1 can activate NF-κB and STAT3 signaling resulting in an increased expression of VEGF, COX-2, c-Myc, and Bcl-xL to induce angiogenesis and cell growth, and inhibit apoptosis [32]. EBER activates the TLR3 signaling pathway resulting in an increased expression of inflammatory cytokines including TNFα and IL-6 in an NPC cell line [33]. Moreover, EBERs are able to modulate the inflammatory response via RIG-I-dependent NF-κB and IRF3 signaling pathways. The expression level of RIG-I in HNE2 cells treated with EBER was correlated with EBER transcription levels in a dose-dependent manner resulting in induction of the inflammatory cytokines, IL-1α, IL-6 and TNF-α. EBER also induces IL-8 production to recruit macrophages to the tumor environment and differentiation of tumor-associated macrophages (TAM) [34].

In sinonasal tumors such as NP and SIP, numbers of immune cells are recruited into the tumor microenvironment. Zhao and colleagues [29] suggested that the increased infiltration of neutrophils, macrophages, CD8+ T cells, and T-reg cells in SIP may affect the activation of cell-mediated innate immune responses and indicate the involvement of viral infection in pathogenesis of SIP. Prominent infiltration and activation of neutrophils and macrophages might induce production of free radicals and proteolytic enzymes and contribute to tissue damage and possibly trigger epithelial cell remodeling [29]. In the present study, we found that inflammation was present in 18.75 and 6.25% of NP and SIP samples, respectively. EBV prevalence was significantly higher in SIP (64.06%) compared to NP (33.75%) tissues and the OR demonstrated that EBV infection significantly increased the risk of SIP development (OR = 3.52; CI = 1.70–7.28). All SIP tissues with chronic inflammation and with sub-acute inflammation were EBV-positive. EBER transcription was found in EBV-positive infiltrating cells within tumor stroma. Given that EBER can induce inflammation in NPC cells, it is possible that EBER transcription in infiltrating cells within the tumor stroma trigger cells in the environment to produce inflammatory mediators, resulting in recruitment and accumulation of inflammatory cells at the site of infection. Proinflammatory cytokines produced by these cells might promote the inflammation condition in EBV-associated NP, SIP, and SNSCC. We mostly found EBV infection in undifferentiated and poorly differentiated SNSCC cases, as well as in SIP cases with mild and severe dysplasia, consistent with previous findings for esophageal carcinomas [35] and nasopharyngeal carcinomas (NPC) [36]. EBV prevalence was also greater in mild, moderate, and severe dysplasia of the oral epithelium [37]. All these data suggest that cellular proliferation and differentiation might be affected by EBV infection.

We found EBV DNA in 50 and 33.33% of the recurrent SNSCC and SIP cases, respectively. There was no statistically significant correlation between EBV infection and disease recurrence. Among reported risk factors for recurrence of SNSCC and SIP are patient behavior, lesion site, staging, malignant association, surgical technique, incomplete removal of the primary tumor, as well as infection with viruses such as HPV [38–40]. Among these, the most important factors seem to be staging, incomplete removal, malignancy, and surgical technique [38, 39, 41, 42]. This evidence suggests that several factors as well as EBV infection are involved in recurrence of SNSCC and SIP.

Moreover, a recent study has shown a high prevalence of EBV (45.5%) in SNSCC, among which only EBV-positive cases developed lymph node or distant metastases [18]. A significantly higher metastasis rate was observed in EBV/LMP1-positive cases. This suggested a strong association between EBV infection and metastasis of SNSCC, leading to the conclusion that presence of EBV in SNSCC tissues might be a risk factor for SNSCC progression [18]. This is consistent with our observation that only EBV-positive cases developed lymph-node metastasis. As mentioned above, EBV oncoproteins such as EBERs are able to recruit inflammatory and other immune cells into the tumor microenvironment [30], and can induce them to produce inflammatory cytokines. IL-1α, IL-6 and TNF-α are inflammatory cytokines induced by EBER via RIG-I activation. Dysregulation of TNF-α production is the factor driving chronic inflammation [43] and able to induce reactive oxygen species (ROS) causing host cell DNA damage, genetic instability and tumorigenesis [44]. In addition, dysregulation of TNF-α production can induce expression of matrix metalloproteinase-9 (MMP-9), which is an enzyme involved in degradation of extracellular matrix and promotion of tumor metastasis [45]. IL-6 is produced by several immune cell types as well as tumor cells and is able to act in pro- and anti-inflammatory roles that support cancer cell proliferation, survival, metastasis, angiogenesis and immune evasion [46]. In esophageal adenocarcinoma, IL-6 is secreted by cancer-associated fibroblasts (found in the tumor microenvironment), induces epithelial-to-mesenchymal transition of esophageal adenocarcinoma cell lines OE19 and OE33, and induces cell migration [47]. According to the literature, cytokines and inflammatory mediators produced by several immune cells within the tumor microenvironment and infected or tumor cells can have both anti- and pro-inflammatory roles and act against or in support of tumorigenesis. EBERs are expressed in all infected cells and are able to release and trigger the cells within the surrounding microenvironment. It might be that cytokine expression induced by EBER in the tumor microenvironment is the factor that supports tumorigenesis and progression of EBV-associated NP, SIP and SNSCC.

## Conclusions

We found EBV DNA in all categories of tissue samples using SYBR green real-time PCR. Only a few infiltrating lymphocytes were EBV-positive: tumor cells were not found to be infected. Prevalence of EBV DNA was significantly greater in SIP tissues and somewhat greater (non-significantly) in SNSCC tissues compared to NP samples. Therefore, it is inconclusive whether EBV is a causative agent of SIP, but it is possible that EBV is involved in tumorigenesis and malignant transformation of SIP by inducing inflammation and creating a microenvironment suitable for tumor development. However, the association and the roles of EBV infection in nasal tumors need to be further elucidated.

## Data Availability

The datasets during and/or analyzed during the current study available from the corresponding author on reasonable request.
